# State of affairs in use of steroids in diffuse intrinsic pontine glioma: an international survey and a review of the literature

**DOI:** 10.1007/s11060-016-2141-x

**Published:** 2016-05-13

**Authors:** Sophie E. M. Veldhuijzen van Zanten, Ofelia Cruz, Gertjan J. L. Kaspers, Darren R. Hargrave, Dannis G. van Vuurden, SIOPE DIPG Network

**Affiliations:** Division of Oncology/Haematology, Department of Paediatrics, VU University Medical Center (VUmc), De Boelelaan 1118, Room KTC4.027, 1081 HZ Amsterdam, The Netherlands; Department of Paediatric Oncology, Hospital Sant Joan de Déu (HSJD), Passeig Sant Joan de Déu, 2, Esplugues De Llobregat, 08950 Barcelona, Spain; Princess Máxima Center of Pediatric Oncology, Lundlaan 6, 3584 EA Utrecht, The Netherlands; Paediatric Oncology Unit, Great Ormond Street Hospital (GOSH), Great Ormond Street, London, WC1N 3JH UK

**Keywords:** Diffuse Intrinsic Pontine Glioma (DIPG), Pediatric oncology, Quality of life (QoL), Steroids, Side effects

## Abstract

Children diagnosed with diffuse intrinsic pontine glioma (DIPG) face a dismal prognosis, with severe neurologic deterioration and inevitable death at a median of 9 months from diagnosis. Steroids are widely prescribed as supportive or palliative treatment although they are known to cause severe side effects that may reduce the quality of life. This study aims to review the current knowledge on, and use of, steroids in DIPG patients. A global questionnaire-study among health care professionals was performed to ascertain information on the current (multi-)institutional and (multi-)national use of steroids, the availability of clinical guidelines, and the need for improvements in prescribing steroids to DIPG patients. In addition, an extensive literature search was performed to review studies investigating steroids in pediatric brain tumor patients. From 150 responding health care professionals, only 7 % had clinical guidelines. The use of steroids was heterogeneous and over 85 % of respondents reported serious side effects. Fourteen articles, with low level of evidence, described the use of steroids in pediatric brain tumor patients. Clinical trials investigating optimal dose or regimen were lacking. This study is a first inventory of the availability of evidence-based information and clinical guidelines, and the current attitude towards the use of steroids in DIPG patients. To date, the risk–benefit ratio of steroids in this disease is yet to be determined. We emphasize the need for clinical trials resulting in guidelines on steroids, and possibly alternative drugs, to optimize the quality of care and quality of life of DIPG patients.

**Electronic supplementary material:**

The online version of this article (doi:10.1007/s11060-016-2141-x) contains supplementary material, which is available to authorized users.

## Introduction

Diffuse intrinsic pontine glioma (DIPG) is a childhood brain tumor that grows diffusely in between the critical structures of the brainstem. Patients have a two-year survival rate of less than 10 % [1]. Given the poor prognosis and lack of effective treatment options, maintenance of a good quality of life for as long as possible should be a major goal in the management approach of DIPG patients. Steroids are widely prescribed as supportive or palliative treatment. They are, however, well known to cause numerous side effects, which in turn may comprise the patient’s quality of life. To date, little research into the risk–benefit ratio and use of steroids in DIPG patients has been performed.

Symptoms in DIPG patients are a result of either direct tumor invasion and destruction of the critical brainstem structures, or tumor- or edema induced compression. Elevated tissue pressure by tumor and edema results in the classical neurological triad of cranial nerve deficits, extremity weakness and ataxia at time of diagnosis [2]. As the tumor grows, the increase in clinical symptoms inexorably leads to a further decrease in the quality of life, and tumor-induced compression and destruction of critical structures for autonomic functioning inside the brainstem eventually heralds death. Edema formation or bleeding within the tumor may accelerate this deterioration. Steroids are used to temporarily relieve symptoms caused by peritumoral edema and are thought to prolong life at end-stage disease.

Although steroids have proven to be very effective in reducing peritumoral edema, they are known to cause substantial side effects, especially with continuous use. Side effects include sleep disorders, mood and behavioral changes, insatiable appetite, weight gain and Cushing’s syndrome, often accompanied by disfiguring striae and a ‘moon face’, completely changing the appearance of the child [3]. These side effects substantially compromise quality of life. It is therefore of the utmost importance to weigh the risks and benefits of steroid treatment.

In this study we aim to survey how health care professionals who manage DIPG patients use steroids in daily practice (e.g., which drugs, dosages, duration and schedules). In addition, we provide an evidence-based overview of the current literature on the use of steroids in DIPG and other pediatric brain tumor patients. The ultimate purpose of this study is to provide a needs-assessment, to facilitate the development of a steroid treatment guideline to optimize care and quality of life of DIPG patients.

## Materials and methods

### International online survey

To ascertain information on the current multi-institutional and multi-national use of steroids in DIPG patients, the availability of clinical guidelines, and to learn possible points for improvement in prescribing steroids to DIPG patients, an international survey, using an online questionnaire, was developed and distributed among health care professionals specialized in DIPG.

The survey assessed the institutional use of steroids; e.g. prescribed drugs (type of steroid), dose and dosing schedule, route of administration, duration of steroid therapy, time of initiation during disease course, and tapering regimens. Different types of steroid doses were converted to dexamethasone equivalents in mg per m^2^ per day by multiplying with 0.1875 for methylprednisolone, 0.15 for prednisone/prednisolone and 1.25 for betamethasone. Doses given in mg/kg/day were converted by multiplication with 30 [4]. An average prescribed dose was thus calculated for each respondent. For tapering regimens, the duration over which the dose was reduced, and to what extent, was analyzed. No differentiation was made between the use of steroids at the time of diagnosis and/or at the time of palliation. Instead, the respondents were asked a general question on when steroids are usually prescribed during the disease course (e.g. at time of diagnosis, during radiotherapy, at relapse and/or at the terminal phase of the disease). The observed effects and side effects of steroid treatment were ascertained. Availability of local clinical guidelines was asked. Items of this questionnaire may be found in the Supplementary Material.

The survey was distributed world-wide to experts treating children with brain tumors, via the electronic mailing lists of the International Society of Paediatric Oncology Europe (SIOPE) Brain Tumour Group, the International Society of Paediatric Neuro-oncology (ISPNO) and the International Brain Tumor Alliance (IBTA). Two weeks after the initial distribution, a reminder was sent. The survey was also promoted in the online newsletter from the IBTA.

### Statistical analysis

The data from the survey were analyzed by descriptive statistical methods using IBM SPSS Statistics for Windows, Version 20.0 (Armonk, NY: IBM Corp. Released 2011). Nominal categorical variables were descriptively analyzed regarding to the observed frequencies. Numerical variables were evaluated using central tendency and dispersion measures.

### Review of the literature

To identify all possible information on the use of steroids in children with brain tumors, a review of the current literature was performed to obtain evidence-based information, especially on clinically studied drugs and dosages, and the duration and/or schedules that have been tested thus far. In advance of the search, it was known that literature on the use of steroids in DIPG patients is scarce. It was therefore our explicit purpose to broaden the search to all pediatric brain tumors, in an aim to find information that is translatable to DIPG patients.

The complete databases of Medline/PubMed, Embase and The Cochrane Library were searched for relevant articles. The search strategy combined controlled and free text words for the target population (e.g. children), the tumor type (e.g. brain tumors), the underlying pathophysiology (e.g. edema) and all types of steroidal drugs. Inclusion criteria were: case reports, clinical studies and literature reviews investigating the use of steroids for pediatric brain tumor patients. The complete search strategy may be found in the supplementary material.

## Results

### Online international questionnaire-study

**Fig. 1:**
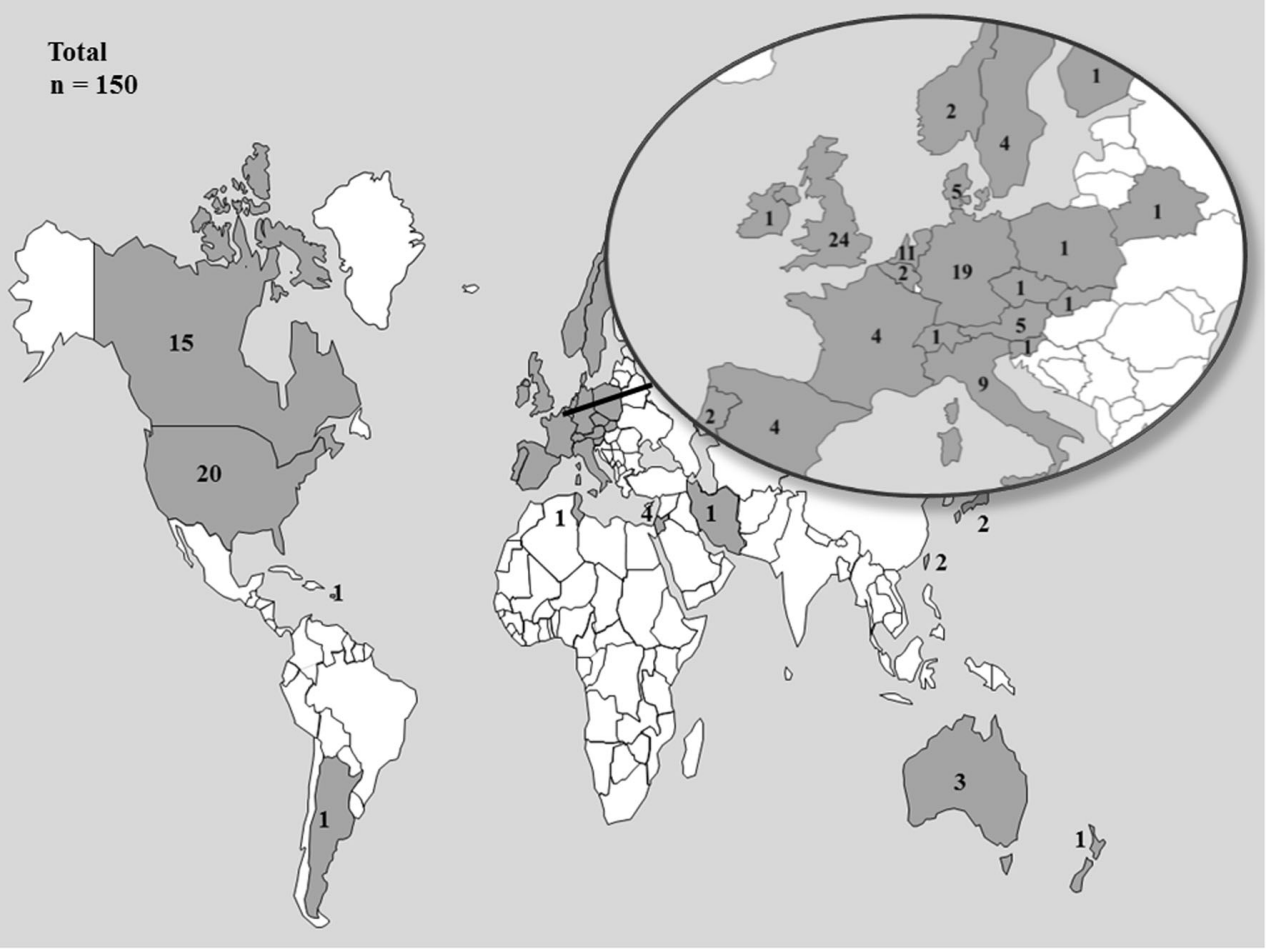
Geographical distribution of respondents to the international online survey

One hundred and fifty health care professionals who manage DIPG patients from 31 countries responded to the online survey (Fig. 1). It was not possible to determine the response rate from the electronic mailing lists of the SIOPE, ISPNO and the IBTA, as (1) it was unclear how many professionals were included, (2) these lists also include professionals not directly involved in the treatment of DIPG patients, and (3) professionals were also asked to forward this invitation to the survey to colleagues within their institution and/or national groups. In total, professionals from 20 European and 11 non-European completed the survey. Most respondents were pediatric oncologists (121, 81 %). Others included radiotherapists, pediatric neurologists or pediatric neurosurgeons (supplementary material). The health care professionals answering the questionnaire treated a median of two DIPG patients per year (range 1–25 patients). There were 11 professionals, working in larger volume centers located in the Italy (n = 3), the United Kingdom (n = 1), the United States of America (n = 6) and Argentina (n = 1) who indicated treating eight or more DIPG patients each year.

The vast majority, 93 % of respondents, responded that no specific guideline for the prescription of steroids was used in their institution. Guidelines were available in all but one of the larger volume centers. Sixty-seven percent of those that used a guideline were willing to share this with the community.

**Fig. 2:**
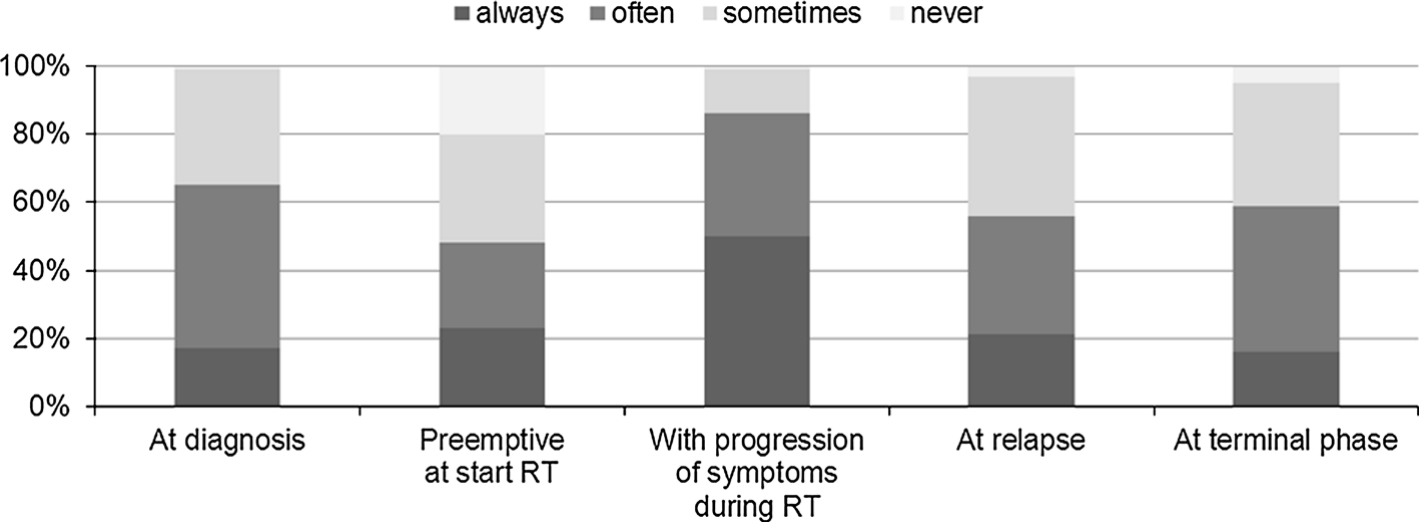
The pattern of steroid prescription during the disease course

Steroid therapy was in most cases initiated by a pediatric oncologist (82 %) or radiotherapist (18 %). Neurosurgeons, general pediatricians, family doctors or parents are less likely to lead on the initiation (supplementary material). Steroids are prescribed during the entire disease course (e.g. at time of diagnosis, during radiotherapy, at relapse and at the terminal phase of the disease; Fig. 2), without a clear pattern, but prescription is mainly driven by clinical symptoms

**Fig. 3:**
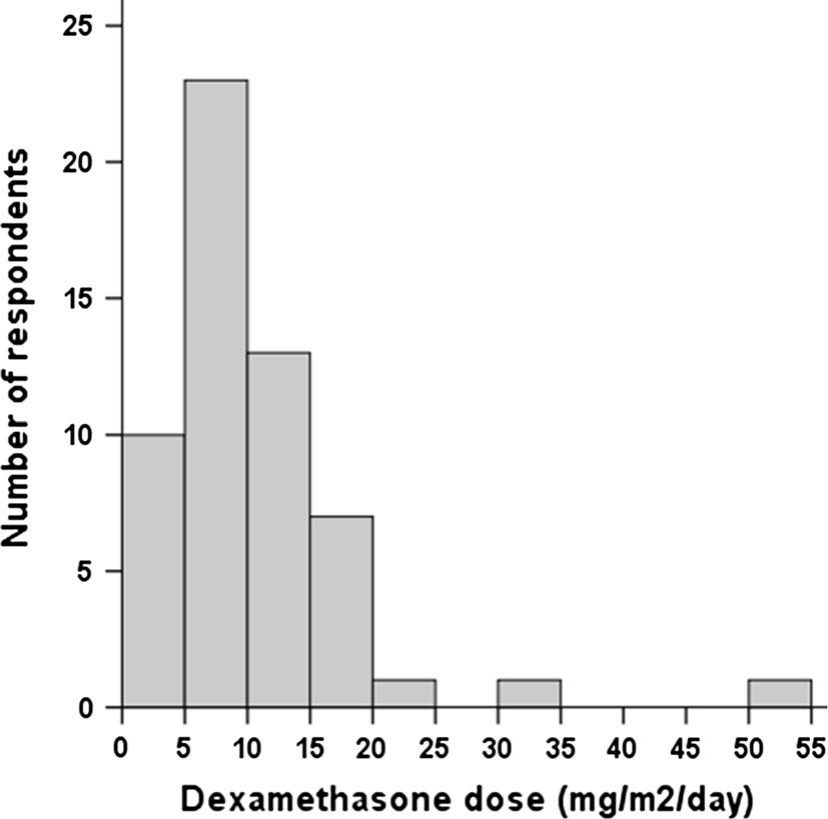
Distribution of steroid dosage. Starting dose converted to dexamethasone equivalents

**Fig. 4:**
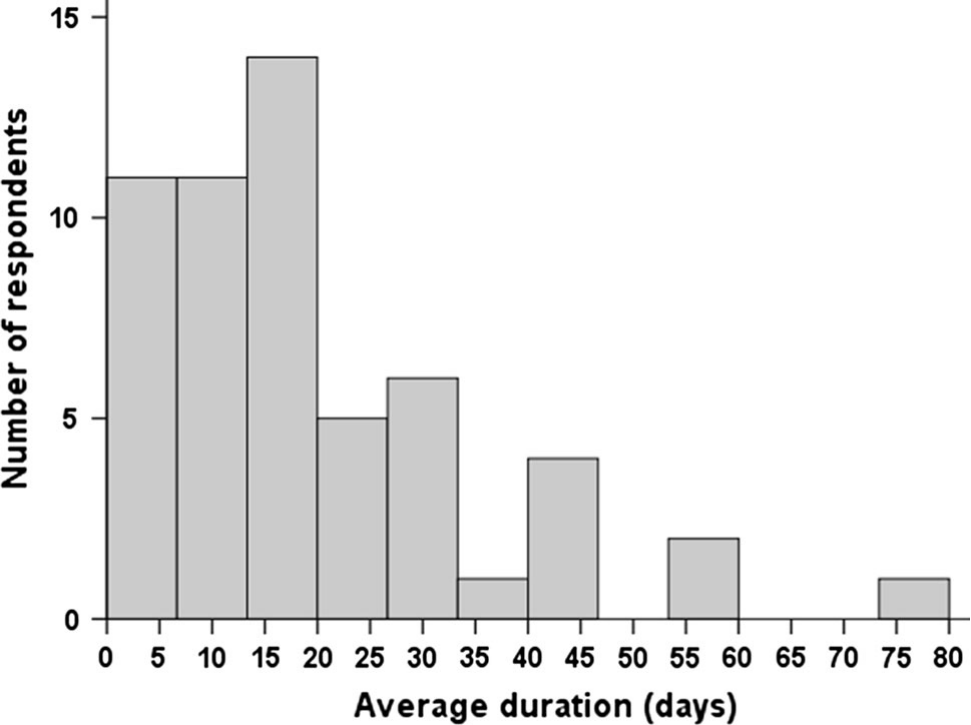
Distribution of steroid treatment duration

The types of steroids used are dexamethasone, methylprednisolone, betamethasone, prednisolone and prednisone. The vast majority of respondents (91 %) prescribe dexamethasone (supplementary material). The most common route of administration is oral, which is rarely combined or interspersed with intravenous administration (supplementary material). Heterogeneous dosing was observed, both in dose as in duration. Converted doses varied from 1.5 to 52.5 mg/m^2^/day with a median of 8.5 mg/m^2^/day (Fig. 3). Steroid prescription was slightly less heterogeneous in European countries and in larger volume centers, ranging from 2.25 to 22.5 mg/m^2^/day (median 8 mg/m^2^/day) and 2.25–30 mg/m^2^/day (median 8.5 mg/m^2^/day), respectively. The duration varied from 3 to 75 days with a median of 15 days (Fig. 4). Stopping or tapering regimens also varied; most respondents (75 %) always taper, whereas 9 % of respondents do not taper steroid therapy but stop instantly and 16 % taper if the steroids were prescribed over a ‘longer period of time’ (i.e. prescription ranging from >5 to >14 days). The tapering regimen duration varied from 1 to 35 days and the dose reduction per step varied from 10 to 50 % per day, or 0.1–4.0 mg/m^2^/day. For tapering and duration there was no difference observed between larger or smaller volume centers.

**Fig. 5:**
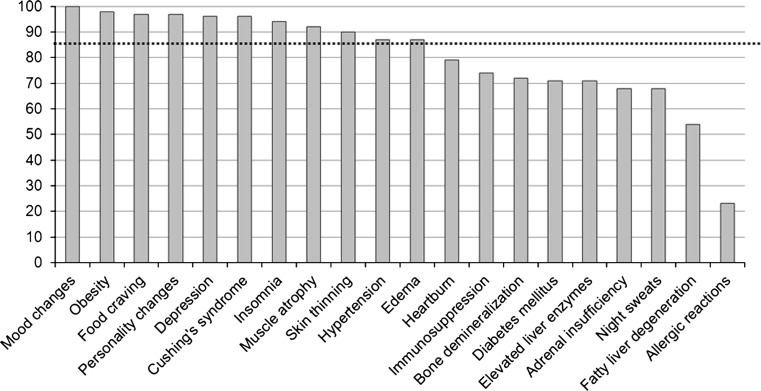
Reported steroid side effects. *Dotted line* reported by >85 % of respondents

Commonly observed steroid side effects, as described by more than 85 % of respondents were: mood changes, obesity, food craving, personality changes, depression, Cushing’s syndrome, insomnia, muscle atrophy, skin thinning, hypertension and edema (Fig. 5). Long-term steroid-induced side effects of steroid administration were less often described; immunosuppression (e.g. increased infections), bone demineralization, diabetes mellitus, elevated liver enzymes, adrenal insufficiency and fatty liver degeneration. Occurrence of allergic reactions along with steroid administration was described by 23 % of the respondents.

Sixty-eight percent of respondents believe that steroids are of great help in management of symptoms in DIPG patients, although it is acknowledged that there is a tight balance between benefits and side effects of steroids in these patients. Forty-six percent of respondents hold the opinion that the observed side effects do not outweigh the established efficacy and 77 % of respondents state that steroid alternatives are urgently needed. To overcome or reduce side effects, 16 % of respondents use alternatives to steroids. Reported alternatives included bevacizumab, boswelic acids, osmotic diuretics, mannitol, acetazolamide, celecoxib, and high dose bicarbonate (supplementary material). More than 70 % of the respondents agree with the statement that steroid regimens are to be investigated in DIPG patients and that an international DIPG steroid guideline should be developed for these patients.

### Literature review

The literature search resulted in 844 records. Fourteen papers were selected for full text analysis [3, 5–17]. Excluded papers investigated the use of steroids in animals, adults, and children with steroid use for other indications (i.e. asthma, nephrotic syndrome, leukemia, trauma, meningitis, etc.).

Table 1 shows the complete list of studies investigating or describing the use of steroids in pediatric brain tumor patients, and their level of evidence. Full text analysis, revealed that most papers describe the use of dexamethasone, with doses ranging from 0.15 mg/kg/day (4.5 mg/m^2^/day) [11] to 2.0 mg/kg/day (60 mg/m^2^/day) [15]. Only four studies describe the effects of steroid use [5, 10, 12, 14]. Table 2 shows the side effects of steroid use that were reported in the papers.

**Table 1:** Result of the literature search

Ref. nr.	Year	Author	Study type	Patient type	Disease	What was reported	Level of evidence [24]
[5]	1986	Schmid	Retrospective analysis	38 adults/23 children	Fossa posterior tumors	Effect of steroids	2B
[6]	1988	Freeman	Single arm Phase 1	34 children (aged 3–21)	Brain stem tumors	Side effects of steroids	4
[7]	1991	Freeman	Single arm Phase 1	57 children (aged 3–21)	Brain stem tumors	Side effects of steroids	4
[8]	1995	Toftegaard	Case report	15 year-old girl	Brain tumor	Side effect of steroids	5
[3]	1997	Glaser	Descriptive	62 children	CNS tumors	Side effects of steroids	2C
[9]	1998	Wolff	Retrospective analysis	20 children	Brain tumors	Side effects of steroids	2B
[10]	2000	Mursch	Retrospective analysis	55 children	Brain stem tumors	Effect of steroids	2B
[11]	2002	Edelbauer	Double arm study	60 children (aged 1–18)	Brain tumors	Side effects of steroids	1B
[12]	2008	Mallur	Case report	5 year-old boy	JPA	Effect of steroids	5
[13]	2010	Broniscer	Single arm Phase 1	21 children (aged 2–20)	DIPG	Side effects of steroids	4
[14]	2010	Meyzer	Case report	10 year-old boy	Oligodendroglioma	Effect of steroids	5
[15]	2011	Beltran	Single arm	15 children (aged 2–13)	DIPG	Side effects of steroids	1B
[16]	2012	Wheeler	Case report	12 year-old boy	Supratentorial GBM	Side effects of steroids	5
[17]	2012	Yamasaki	Survey	Children	Cancer and brain tumours	Side effects of steroids	5

Complete list of studies investigating or describing the use of steroids in pediatric brain tumor patients, and their level of evidence

*Ref. nr.* reference number, *JPA* Juvenile pilocytic astrocytoma, *GBM* Glioblastoma multiforme

**Table 2:** Reported side effects of steroid use in pediatric brain tumor patients

Reference number:	[6]	[7]	[8]	[3]	[9]	[11]	[14]	[16]	[17]	[18]
Personality-/mental-/behavioral-/mood changes		*		*					*	
Gastrointestinal (peptic) ulcer, hemorrhage and perforation/gastritis				*						*
Increased/insatiable appetite				*						
Moon face/obesity/weight gain				*				*	*	*
Altered body habitus				*						
Iatrogenic cushing syndrome									*	
Abnormal glucose tolerance/elevated blood glucose		*							*	*
Diabetic ketoacidosis	*									
Osteoporosis		*		*						*
Aseptic bone necrosis										*
Hypertension		*					*		*	*
Posterior reversible encephalopathy syndrome (PRES)							*			
Glaucoma										*
Addisonian crisis risk up to 1 year after cessation				*						
(Proximal) myopathy				*					*	
Cataract				*						
Reduced permeability of BBB to chemotherapeutic agents										
Skin atrophy/abdominal striae (striae sistensae)/interference with wound healing									*	
Glucocorticoid-induced and vasopressin-resistant polyuria			*							
Hepatotoxicity					*					
*Glutamate oxalacetate transaminase (GOT)					*					
*Glutamate pyrovate transaminase (GPT)					*					
Altered immune response/immunosuppression/opportunistic infections	*	*		*		*				*
*Inhibition of the transcription of IL-4/IFN-c/TNF-a/IL-3/IL-5						*				
*Enhancement of in vitro IL-4 production in PBMCs						*				
*Shift towards the Th2 humoral immune response						*				
*IgE antibody production with danger of anaphylactic reactions						*				
*Pneumocystis jiroveci pneumonia (PJP), formerly known as Pneumocystis carinii pneumonia (PCP)	*									
*Disseminated varicella	*									
*Mucocutaneous Candidiasis				*						
Significant decrease in Quality of Life									*	

## Discussion

Our extensive worldwide survey with 150 respondents from all over the world provides a good overview of the current use of steroids in DIPG patients. This international survey uncovers an *absence of clinical guidelines*, a strikingly *heterogeneous use of steroids in DIPG patients*, and a *significant amount of reported side effects*. A meager 11 (7 %) of the surveyed health care professionals indicated to have a clinical guideline available. These guidelines were mainly used in larger volume centers. Steroids are prescribed throughout the entire DIPG disease course and almost all known side effects from steroid use occur in over 50 % of patients. Oral dexamethasone is by far the most common prescription, but the dosages, duration and tapering regiments showed such a wide variety that the current data are insufficient to develop clinical recommendations. In some cases it was even questionable whether the responses reflect the reality, with respondents indicating to dose as low as 1.5 mg/m^2^/day up to as high as 52.5 mg/m^2^/day. A limitation of the survey, however, is the fact that it did not separately address steroid prescription at the time of diagnosis from prescription at time of disease progression. Furthermore, respondents were not asked whether steroids were prescribed in the context of a clinical trial. This may have influenced the results of the obtained dose-range and should be taken into account in future studies. Finally, health care professionals indicate that both optimization of steroid therapy and exploration of alternative options to steroid therapy are urgently needed in an aim to provide better supportive and palliative care for patients suffering from a DIPG.

For children with DIPG, treatment is essentially palliative and quality of life is of paramount importance [18]. Although steroids are widely prescribed, we show that there is a striking lack of literature and evidence available on this important topic. Only 14 studies report on the effects and/or side effects of steroid use in pediatric brain tumor patients. Only four studies focus on DIPG patients specifically [6, 7, 13, 15]. Most articles have low level of evidence, do not specifically study the use of steroids and more importantly, clinical trials determining the optimal drugs, dosage and schedules are lacking. The dosages that were found in the literature show a great diversity when converted to dexamethasone equivalents: from a 4 mg single dose up to a cumulative 24-h dose of 66 mg/m^2^ in young patients undergoing brainstem surgery/biopsy [8, 10]. The observed diversity was partly related to the indication for steroid therapy. Merely four articles report on the positive effects of steroids, which in most cases is a reduction of clinical symptoms [5, 10, 12, 14] and in one case changes observed by MR-imaging [14]. One article reports a possible negative effect of steroids: a decrease of the blood–brain barrier (BBB) permeability to (water soluble) cytotoxic agents aimed at treating the tumor [3]. The possibly more important negative effect of immunosuppression, namely a reduction of anti-tumor immunogenicity, is not mentioned in any of the articles from the literature search [19]. Twelve articles report on steroid side effects, but only two studies performed active prospective registration [11, 15]. To conclude, in literature there currently is no high-level evidence on the (side) effects and optimum steroidal drugs, dosages, duration and schedules for children suffering DIPG.

The results of our study show that clinical guidelines on the use of steroids are urgently needed. To substantiate these guidelines, further research is warranted, investigating both steroid schedules, for instance by means of randomizing between short courses of high dosages and prolonged use of lower dosages, as well as studies into more patient friendly alternatives to steroids, such as bevacizumab, boswelic acids, and possibly corticotropin-releasing factor (hCRF) analogue corticorelin acetate [18, 20–22], or palliative re-irradiation [23]. Prospective (randomized) clinical trials specifically developed for DIPG patients are a prerequisite, since edema-related symptoms in these patients occur more frequently and rapidly than in patients suffering from other types of brain tumors. Also, the balance of the risk–benefit ratio may be different in DIPG patients: as there is no curative treatment yet, it should be judiciously considered whether one should extend life by the use of steroids, whilst incurring side effects that decrease the quality of life. Outcome measures such as performance score and quality of life should therefore be carefully recorded, in parallel to recording of BMI and observed side effects. In the design of a prospective clinical trial, there will be a number of challenges, such as the rarity of the disease resulting in problems to power the trial or to address the many variations of use obtained from our worldwide survey, but possibly also breaking well-established individual-experience based practices that oncologists may not be willing to stop, and finally human subjects protection concerns related to this vulnerable group of patients. We therefore would recommend commencing the prospective registration of current practices in the prescription of steroids in DIPG patients. Such aims can be facilitated by the recent establishment of the SIOPE DIPG registry (http://www.dipgregistry.eu) by the SIOPE DIPG network, and the International DIPG registry (http://www.dipgregistry.org). Prospective registration should include all items from the survey, and should be designed for longitudinal registration (e.g. during all phases of the disease course). Prophylaxis during prolonged steroid prescription, such as for Pneumocystis jiroveci pneumonia (PJP), should also be assessed. In order to allow the collection of uniform data, standardized case report forms will be developed and included in the registries shortly.

To conclude, this study has provided insight in the current wide variety of steroid use in DIPG. We believe that prospective registration of current practices is a prerequisite step in advance of the development of a multinational clinical trial and a future guideline. Determining the risk–benefit ratio of steroid use will be challenging, but is needed to optimize supportive care and quality of life for patients suffering from DIPG.

## Electronic supplementary material

Below is the link to the electronic supplementary material.

Supplementary material 1 (DOCX 28 kb)

Supplementary material 2 (DOCX 121 kb)

Supplementary material 3 (PDF 458 kb)
