# Supplementary material for: State of affairs in use of steroids in diffuse intrinsic pontine glioma: an international survey and a review of the literature
Source: J Neurooncol. 2016 May 13;128:387–94. doi: 10.1007/s11060-016-2141-x (PMC4901114; doi:10.1007/s11060-016-2141-x)
Supplement: Supplementary file 1 — Supplementary material 1 (DOCX 28 kb) [file 11060_2016_2141_MOESM1_ESM.docx]

**SUPPLEMENTARY MATERIAL – STEROID SURVEY RESULTS**

**State of affairs in use of steroids in diffuse intrinsic pontine glioma: An international survey and a review of the literature**

*Journal of Neuro-Oncology*

Veldhuijzen van Zanten SEM (corresponding author, VU University Medical Center/Division of Paediatric Oncology-Haematology, s.veldhuijzen@vumc.nl), Cruz O, Kaspers GJL, Hargrave D, Van Vuurden DG on behalf of the SIOPE DIPG Network

| **Number of respondents** | **Overall** |
| --- | --- |
| See Figure 1 | 150 |
|  |  |
| **What is your profession?** | **(%)** |
| * Pediatric Oncologist | 81 |
| * Radiotherapist | 6 |
| * Pediatric Neurologist | 5 |
| * Pediatric Neuro-surgeon | 3 |
| * Pediatrician | 1 |
| * Oncologist | 1 |
| * Pediatric palliative medicine consultant | 1 |
| * Nurse*^*^* | 1 |
| * Pathologist*^*^* | 1 |
| *^*^ did not participate as prescriber of steroids, but as members of the multidisciplinary team; able to identify adverse effects and tissue changes.* |  |
| **How many DIPG patients do you treat on average each year?** | **Overall** |
| * Median (Q1-Q3) | 2 (1-4) |
| * Min – Max | 1 – 25 |
|  |  |
| **Questions regarding the use of steroid guidelines for DIPG** | **(% yes)** |
| * Do you use a specific steroid guideline in your institution? | 7 |
| * If so, would you be willing to share your guideline with the SIOPE DIPG Network? | 67 |
|  |  |
| **Who leads on the initiation of steroid therapy in your practice? (answer yes/no)** | **(% yes)** |
| * Pediatric oncology team | 82 |
| * Radiotherapy team | 18 |
| * Neurosurgeon | 7 |
| * Child's pediatric team | 3 |
| * Parents | 2 |
| * Child's family doctor | 0 |
|  |  |
| **At what time in the disease course do you prescribe steroids to your DIPG patients?** |  |
| See Figure 2 | 150 |
|  |  |
| **Which steroids (generic name) do you usually prescribe in DIPG patients?** | **(%)** |
| * dexamethasone | 91 |
| * methylprednisolone | 4 |
| * betamethasone | 2 |
| * prednisolone | 2 |
| * prednisone | 1 |
|  |  |
| **How do you administer steroids?** | **(%)** |
| * Oral (p.o.) | 80 |
| * Oral (p.o.) and Intravenous (i.v.) | 14 |
| * Intravenous (i.v.) | 2 |
| * Subcutaneous infusion (csci) | 1 |
| * Oral nasogastric (ng) | 1 |
| * Percutaneous endoscopic gastrostomy (PEG) | 1 |
| * Subcutaneous (SubQ) | 1 |
|  |  |
| **What dose, frequency and tapering regime to you use in DIPG patients?** |  |
| See Figure 3 & 4 |  |
|  |  |
| **Which of the following side effects do you encounter in patients with DIPG?** | **(% yes)** |
| See Figure 5 |  |
|  |  |
| **Do you use alternatives to steroids? If yes, please specify** | **(%)** |
| * Yes | 16 |
| * Please specify: |  |
| *- boswelic acids (or frankincense)* | *n = 8* |
| *- bevacizumab* | *n = 5* |
| *- mannitol* | *n = 3* |
| *- shunt placement* | *n = 2* |
| *- osmotic diuretics* | *n = 1* |
| *- acetazolamide* | *n = 1* |
| *- colecoxib* | *n = 1* |
| *- pain therapy* | *n = 1* |
| *- ondansetron* | *n = 1* |
| *- high dose bicarbonate* | *n = 1* |
|  |  |
| **Do you agree or disagree with each of the following statements? Or are you neutral?** | **(%)** |
| * Steroids are of great help in management of symptoms |  |
| *- Agree* | 68 |
| *- Disagree* | 5 |
| * There is a close balance between effect and side effects of steroids in DIPG |  |
| *- Agree* | 67 |
| *- Disagree* | 15 |
| * The observed side effects outweigh the established efficacy |  |
| *- Agree* | 24 |
| *- Disagree* | 46 |
| * Steroid alternatives are urgently needed |  |
| *- Agree* | 77 |
| *- Disagree* | 8 |
| * Steroid regimens should be investigated in DIPG patients |  |
| *- Agree* | 73 |
| *- Disagree* | 11 |
| * A (European) DIPG steroid guideline should be developed |  |
| *- Agree* | 76 |
| *- Disagree* | 7 |
