# Supplementary material for: State of affairs in use of steroids in diffuse intrinsic pontine glioma: an international survey and a review of the literature
Source: J Neurooncol. 2016 May 13;128:387–94. doi: 10.1007/s11060-016-2141-x (PMC4901114; doi:10.1007/s11060-016-2141-x)
Supplement: Supplementary file 2 — Supplementary material 2 (DOCX 121 kb) [file 11060_2016_2141_MOESM2_ESM.docx]

**SUPPLEMENTARY MATERIAL – SEARCH STRATEGY**

**State of affairs in use of steroids in diffuse intrinsic pontine glioma: An international survey and a review of the literature**

*Journal of Neuro-Oncology*

Veldhuijzen van Zanten SEM (corresponding author, VU University Medical Center/Division of Paediatric Oncology-Haematology, s.veldhuijzen@vumc.nl), Cruz O, Kaspers GJL, Hargrave D, Van Vuurden DG on behalf of the SIOPE DIPG Network

**PubMed**

"Steroids"[Mesh:NoExp] OR "Dexamethasone"[Mesh] OR "Prednisone"[Mesh] OR "Prednisolone"[Mesh] OR "Hydrocortisone"[Mesh] OR "Adrenal Cortex Hormones"[Mesh] OR steroid*:ti,ab OR "Dexamethasone":ti,ab OR "Prednisone":ti,ab OR "Prednisolone":ti,ab OR "Hydrocortisone":ti,ab OR corticosteroid*:ti,ab OR "Adrenal Cortex Hormone":ti,ab OR "Adrenal Cortex Hormones":ti,ab **AND** "Brain Neoplasms"[Mesh] OR "Brain Edema"[Mesh] OR brain neoplasm*:ti,ab OR "peritumoral edema":ti,ab OR "intracranial edema":ti,ab OR (("Central Nervous System"[Mesh:NoExp] OR "Brain"[Mesh] OR brain:ti,ab OR brains:ti,ab OR encephalon:ti,ab OR encephalons:ti,ab OR brainstem:ti,ab OR medulla:ti,ab OR medullary:ti,ab OR mesencephalon:ti,ab OR mesencephalic:ti,ab OR midbrain:ti,ab OR "Pons"[Mesh] OR pons:ti,ab OR pontine:ti,ab OR "Diffuse Intrinsic Pontine Gliomas":ti,ab OR "Diffuse Intrinsic Pontine Glioma":ti,ab OR DIPG:ti,ab ) AND ("Neoplasms"[Mesh] OR Neoplasm:ti,ab OR neoplasms:ti,ab OR neoplasia:ti,ab OR neoplasma:ti,ab OR neoplasmatic:ti,ab OR neoplastic:ti,ab OR cancer:ti,ab OR cancers:ti,ab OR cancerous:ti,ab OR cancereux:ti,ab OR canceration:ti,ab OR cancerisation:ti,ab OR cancerization:ti,ab OR cancerogen*:ti,ab OR cancerology:ti,ab OR cancerologie:ti,ab OR precancerous:ti,ab OR tumour:ti,ab OR tumor:ti,ab OR tumours:ti,ab OR tumors:ti,ab OR tumourous:ti,ab OR tumorous:ti,ab OR carcinom*:ti,ab OR melanom*:ti,ab OR glioma:ti,ab OR gliomas:ti,ab OR glioma’s:ti,ab **AND** child:ti,ab OR children:ti,ab OR children’s:ti,ab childrens:ti,ab OR childhood:ti,ab OR schoolchild*:ti,ab OR infan*:ti,ab OR adolescen*:ti,ab OR pediatri*:ti,ab OR paediatr*:ti,ab OR neonat*:ti,ab OR boy:ti,ab OR boys:ti,ab OR boyhood:ti,ab OR girl:ti,ab OR girls:ti,ab OR girlhood:ti,ab OR youth:ti,ab OR youths:ti,ab OR baby:ti,ab OR babies:ti,ab OR toddler*:ti,ab OR teen:ti,ab OR teens:ti,ab OR teenager*:ti,ab OR newborn*:ti,ab OR postneonat*:ti,ab OR postnat*:ti,ab OR puberty:ti,ab OR preschool*:ti,ab OR suckling*:ti,ab OR picu:ti,ab OR nicu:ti,ab OR offspring:ti,ab OR minor:ti,ab OR minors:ti,ab

**EMBASE**

'steroid'/de OR 'dexamethasone'/exp OR 'prednisone'/exp OR 'prednisolone'/exp OR 'hydrocortisone'/exp OR steroid*:ti,ab OR Dexamethasone:ti,ab OR Prednisone:ti,ab OR Prednisolone:ti,ab OR Hydrocortisone:ti,ab **AND** 'brain tumor'/exp OR 'brain edema'/exp OR 'brain neoplasm':ti,ab OR 'brain neoplasms':ti,ab OR 'brain tumor':ti,ab OR 'brain tumors':ti,ab OR 'brain tumour':ti,ab OR 'brain tumours':ti,ab OR 'brain cancer':ti,ab OR 'brain cancers':ti,ab OR 'brain stem':ti,ab OR brainstem:ti,ab OR pons:ti,ab OR pontine:ti,ab OR DIPG:ti,ab OR 'peritumoral edema':ti,ab OR 'brain edema':ti,ab OR 'intracranial edema':ti,ab **AND** child*:ti,ab OR schoolchild*:ti,ab OR infan*:ti,ab OR adolescen*:ti,ab OR pediatri*:ti,ab OR paediatr*:ti,ab OR neonat*:ti,ab OR boy:ti,ab OR boys:ti,ab OR boyhood:ti,ab OR girl:ti,ab OR girls:ti,ab OR girlhood:ti,ab OR youth:ti,ab OR youths:ti,ab OR baby:ti,ab OR babies:ti,ab OR toddler*:ti,ab OR teen:ti,ab OR teens:ti,ab OR teenager*:ti,ab OR newborn*:ti,ab OR postneonat*:ti,ab OR postnat*:ti,ab OR puberty:ti,ab OR preschool*:ti,ab OR suckling*:ti,ab OR picu:ti,ab OR nicu:ti,ab OR offspring:ti,ab OR minor:ti,ab OR minors:ti,ab
