# Supplementary material for: State of affairs in use of steroids in diffuse intrinsic pontine glioma: an international survey and a review of the literature
Source: J Neurooncol. 2016 May 13;128:387–94. doi: 10.1007/s11060-016-2141-x (PMC4901114; doi:10.1007/s11060-016-2141-x)
Supplement: Supplementary file 3 — Supplementary material 3 (PDF 458 kb) [file 11060_2016_2141_MOESM3_ESM.pdf]

Online steroid survey

SIOP Europe

http://www.thesistools.com/web/?id=357215

ThesisTools
Create and distribute your online survey for free at www.thesistools.com

SIOPe DIPG Network - Steroids Survey

Welcome to the DIPG Network Steroids Survey.

With this survey we inventorize current practise of using steroids in the treatment of children with a DIPG, among physicians treating DIPG patients in non-EU countries

Password: ••••••••••••

Start

ThesisTools
Create and distribute your online survey for free at www.thesistools.com

VU university medical center

SIOPE DIPG network

1.

In which European country do you work?

-- please choose --

2.

At which institution do you practice (name, city)?

3.

What is your profession?*

- Paediatric Oncologist
- Paediatric Neurologist
- Radiotherapist
- Other, please specify:

4.

How many DIPG patients do you treat on average per year?*

5.

Do you use a specific steroid guideline in your institution?

- No
- Yes

6.

If so, would you be willing to share your guideline with the SIOPe DIPG Network?*

- Yes (please provide e-mail address for contact):
- No
- Don't use a guideline

7.

Who/what usually leads on the initiation of steroid therapy in your practise?

- ☐ Parents request
- ☐ Childs family doctor
- ☐ Childs paediatric team
- ☐ Paediatric Oncology team
- ☐ Radiotherapy team
- ☐ Other, please specify: ____

8.

At what time in the disease course do you prescribe steroids to your DIPG patients?

	Always	Often	Sometimes	Never
At diagnosis	○	○	○	○
Preemptive at start radiotherapy	○	○	○	○
During radiotherapy with progression of symptoms	○	○	○	○
At relapse	○	○	○	○
At terminal phase of disease	○	○	○	○

9.

Which steroids do you usually prescribe in DIPG patients and how do you administer?

Generic Name (e.g. dexamethasone)	
Brand Name (e.g. Decadron)	
Route of administration (e.g. oral, intravenous)	

10.

What dose, frequency and tapering regime do you use in DIPG patients?

Starting dose (specify per m2/day or per kg/day)	
How many times per day	
Continuing dose (specify per m2/day or per kg/day)	
How many times per day	
Total average duration of steroid treatment (days)	
Upon discontinuation of steroid therapy, do you taper or stop immediately?	
If tapering, please describe tapering schedule	

11.

Any other remarks on steroid regime in DIPG patients?

12.

Which of the following side-effects do you encounter in patients with DIPG?

	Always	Often	Sometimes	Never
Immunesuppression, increased infections	○	○	○	○
Adrenal insufficiency	○	○	○	○
Cushing's syndrome	○	○	○	○
Obesity	○	○	○	○
Mood changes	○	○	○	○
Personality changes	○	○	○	○
Food craving	○	○	○	○
Depression	○	○	○	○
Insomnia	○	○	○	○

13.

Do you use alternatives to steroids?

- No
- Yes, please specify:

14.

How strongly do you agree or disagree with each of the following statements?

	Fully agree				Fully disagree
Steroids are of great help in management of symptoms	○	○	○	○	○
There is a close balance between effect and side effects of steroids in DIPG	○	○	○	○	○
The observed side effects outweigh the established efficacy	○	○	○	○	○
Steroid alternatives are urgently needed	○	○	○	○	○
Steroid regimens should be investigated in DIPG patients	○	○	○	○	○
A European DIPG steroid guideline should be developed	○	○	○	○	○

15.

Do you have additional comments relating to steroids use in children with DIPG?
